# Sex Hormones and Their Receptors Regulate Liver Energy Homeostasis

**DOI:** 10.1155/2015/294278

**Published:** 2015-09-27

**Authors:** Minqian Shen, Haifei Shi

**Affiliations:** Cell, Molecular, and Structural Biology, Department of Biology, Miami University, 700 E. High Street, Oxford, OH 45056, USA

## Abstract

The liver is one of the most essential organs involved in the regulation of energy homeostasis. Hepatic steatosis, a major manifestation of metabolic syndrome, is associated with imbalance between lipid formation and breakdown, glucose production and catabolism, and cholesterol synthesis and secretion. Epidemiological studies show sex difference in the prevalence in fatty liver disease and suggest that sex hormones may play vital roles in regulating hepatic steatosis. In this review, we summarize current literature and discuss the role of estrogens and androgens and the mechanisms through which estrogen receptors and androgen receptors regulate lipid and glucose metabolism in the liver. In females, estradiol regulates liver metabolism via estrogen receptors by decreasing lipogenesis, gluconeogenesis, and fatty acid uptake, while enhancing lipolysis, cholesterol secretion, and glucose catabolism. In males, testosterone works via androgen receptors to increase insulin receptor expression and glycogen synthesis, decrease glucose uptake and lipogenesis, and promote cholesterol storage in the liver. These recent integrated concepts suggest that sex hormone receptors could be potential promising targets for the prevention of hepatic steatosis.

## 1. Introduction

Obesity rapidly becomes a worldwide epidemic disease with increased risk of cardiovascular diseases, type 2 diabetes mellitus, and metabolic syndrome [1]. Metabolic syndrome is characterized by increased visceral adiposity, hyperlipidemia, insulin resistance, and hypertension [2]. The liver is the largest visceral organ for maintaining homeostasis in glucose, lipid, and protein. Hepatic steatosis is characterized by massive fat accumulation in the liver and thus is strongly related to several features of metabolic syndrome, including hyperlipidemia and insulin resistance [3]. Indeed, reduction or loss of insulin action in the liver leads to abnormally increased hepatic gluconeogenesis, glucose production, and lipogenesis, as well as decreased insulin clearance, hepatic glucose uptake, and lipolysis, consequently resulting in dyslipidemia [4].

Age and sex are physiologic factors that have strong association with the prevalence and features of metabolic syndrome. The state of estrogen deficiency as seen in postmenopausal women and the state of androgen deficiency as seen in aging men predispose older population to the metabolic syndrome and associated diabetes and cardiovascular diseases, indicating that sex hormones play important roles in regulating energy metabolism [5, 6]. Nonalcoholic fatty liver disease (NAFLD) disproportionally affects people with obesity, diabetes with insulin resistance, and dyslipidemia [7–9]. The prevalence of NAFLD varies among ethnicities, with the highest prevalence in Hispanics, correlated with the high prevalence of obesity and insulin resistance in this ethnic group, compared to whites and blacks [10]. Similar to the incidence of metabolic syndrome, the frequency of NAFLD varies between genders, with greater prevalence in men than in women among whites (42% in white men versus 24% in white women) but not in other ethnicities [10]. This is consistent with another epidemiology study showing that the rate of NAFLD is a little higher in men than in women with all ethnicities combined [7]. Interestingly, NAFLD is twice as common in postmenopausal women as in premenopausal women whose estrogen levels are higher than postmenopausal women [7, 11], which suggests the protective role of estrogens in NAFLD [12, 13].

In general, androgens are considered as hormones of the male sex due to their masculinizing effects and their roles in regulating male sexual behavior, whereas estrogens are considered as hormones of the female sex due to their roles in regulating female reproductive physiology and behaviors, although all sex hormones are present in both males and females, albeit at different levels between these two sexes. The most important biologically relevant forms of estrogens and androgens in humans are estradiol (E2) and testosterone, respectively. Understanding of how estrogens and androgens regulate energy metabolism via their receptors may shed light on potential pharmaceutical applications. In the present review, we discuss the roles of estrogens and androgens in regulating liver glucose and lipid homeostasis in rodents and humans. We also deliberate the distinct, important effects of estrogen receptors (ERs) and androgen receptors (ARs) on the regulation of liver metabolism.

## 2. The Role of Estrogens in Regulating Liver Energy Homeostasis

### 2.1. Estrogen Signaling

In both males and females, E2 is derived from the aromatization of testosterone. In premenopausal women, E2 is mainly synthesized from cholesterol in the ovaries, with E2 concentration being approximately 5 times higher than that in men, while in postmenopausal women E2 is primarily converted from testosterone by aromatase in peripheral tissues, such as adipose tissue, adrenal glands, bones, vascular endothelium, and smooth muscle [14], with E2 concentration being similar compared with men (http://www.hemingways.org/GIDinfo/hrt_ref.htm).

Estrogens act on ERs, including classic nuclear receptors ER-*α* and ER-*β*, and membrane-bound receptors, including G protein-coupled ER (GPER, also known as GPR 30) and membrane-associated ER-*α* and ER-*β* variants [15]. All these nuclear and membrane ER subtypes are expressed in the livers of male and female humans and rodents, but at a lower level compared with reproductive organs such as uterus, prostate, testis, ovary, and breast [16–18]. ER-*β* is less abundant in liver cells than ER-*α* [19, 20] and GPER (unpublished observation).

One study by Lax et al. determines levels of ERs in male and female rat livers and reports that the levels of nuclear ERs are not sex dependent but are age dependent, as levels of ERs are similar between male and female rats and vary with the course of life in a comparable manner in males and females [21]. Specifically, levels of ERs in the liver of both male and female rats are the highest during the perinatal period, decline till the onset of puberty, and increase to reach postpubertal peak. Additionally, levels of ERs are maintained as a stable level across the estrous cycles of female rats [21]. Consistently, Eisenfeld group has reported that ER concentration in the rat liver increases evidently at puberty [22]. Ovariectomy (OVX), a procedure that removes ovaries and thus majority of endogenous estrogens, is a suitable preclinical model to study postmenopausal diseases. Liver ER-*α* expression does not change following OVX; however, it significantly increases by E2 treatment at a superphysiological level in rats with OVX, higher than sham-operated rats with intact ovaries and normal levels of endogenous estrogens [23]. These studies indicate that ER-*α* expression in the liver is similar between gonad intact males and females and remains stable in postmenopausal females but could increase following hormone replacement therapy or during puberty. There is no available literature showing changes of expression of ER-*β* and GPER during menstrual period or postmenopausal stage, and these questions remain unknown.

Males also express ERs in the liver, and aromatase metabolizes androgens to generate E2 and other estrogen metabolites locally in many target tissues. A growing body of evidence suggests that estrogens also have important metabolic functions in males. The aromatization of testosterone to E2 is beneficial for preventing intra-abdominal adiposity in men, demonstrated by a clinical study showing increased intra-abdominal fat in men by reduced estrogens due to aromatase inhibition [24]. The effects of estrogens on male and female reproductive organs have been extensively studied, but the beneficial effects of estrogens in nonclassical endocrine targets including the liver are less appreciated. We will discuss how hepatic estrogen signaling via ERs regulates metabolism in male and female animal and human models.

**Figure 1:**
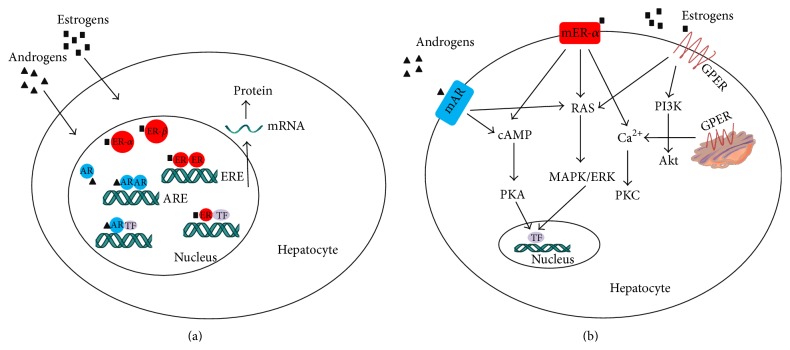
Action of estrogens and androgens via estrogen receptors (ERs) and androgen receptors (ARs) in the liver cells. (a) Genomic effects of estrogens and androgens via nuclear ERs and ARs. (b) Nongenomic effects of estrogens and androgens via membrane-associated ERs and ARs.

Upon estrogen binding, classic estrogen nuclear receptors ER-*α* and ER-*β* form homo- or heterodimers and bind to estrogen response element (ERE) in target gene promoters or to other transcription factors, such as activator protein-1 (AP-1) and stimulating protein-1 [25], to induce expression of target genes. The genomic action following E2-ER binding varies as the level of sex hormone changes. Specifically, the transcriptional activity of ER-*α* alters during the 4-day estrous cycle, demonstrated by using ERE-luciferase reporter mice which have luciferase reporter controlled by activated ERs. The peak of the transcriptional activity of ER-*α* in the liver occurs in proestrus [26], indicating dynamics of ER-*α* transcriptional activity that is possibly modulated by different concentration of estrogens [27]. These findings suggest that liver ER-*α* could recognize the changes in circulating E2 levels and response to reproductive cues during transition of different stages of the estrous cycles and select appropriate genetic programs to adapt the hepatic metabolism to the energy requirements of each stage. Thus, the hepatic ER-*α* could serve as a peripheral coordinator of energy homeostasis. ER-*α* also exists in the form of membrane-associated receptor. There are many lines of evidence showing that the full length ER-*α* and truncated ER-*α* may exert actions via nongenomic signaling which is faster than the classic genomic signaling. Such nongenomic signaling usually involves activation of intracellular second messenger systems, such as protein kinase A (PKA), protein kinase C, and mitogen-activated protein kinase (MAPK)/extracellular signal-regulated protein kinase (ERK) [28–30]. GPER is structurally unrelated to ER-*α* and ER-*β* and is a seven-transmembrane domain G protein-coupled receptor located at the cell membrane and endoplasmic reticulum membrane. GPER is reported to rapidly activate different nongenomic estrogen signaling pathways, including PKA, MAPK/ERK, and phosphoinositide 3-kinase (PI3K) [31] (Figure 1).

### 2.2. Estrogens Regulate Lipid Homeostasis in the Liver

Females, as compared with males, tend to store more energy in subcutaneous fat instead of in visceral fat. The liver is a key visceral organ for controlling energy storage, as the liver has high capacity for lipid transport,* de novo* lipogenesis, lipid oxidation, and lipolysis. Liver steatosis, as seen in the nonalcoholic fatty liver disease (NAFLD), is due to the excess of triglyceride (TG) accumulation within the hepatocytes. Incidence of hepatic steatosis is frequently associated with low levels of high density lipoprotein cholesterol (HDL-C) and high levels of low density lipoprotein cholesterol (LDL-C) in the circulation. Epidemiological studies have showed higher plasma level of LDL-C and lower plasma level of HDL-C in men and postmenopausal women compared with premenopausal women, suggesting that lower circulating estrogen levels may promote fat deposition in the liver [32]. Further evidence is supported by using OVX mouse model combined with pair-feeding between sham operation and OVX groups. Removal of the ovaries and thus the majority of endogenous estrogens in female mice results in increased fat proportion in the liver even when they are pair-fed with the same amount of calories as females with intact ovaries, which indicates the direct role of estrogens in inhibiting lipogenesis in the liver, rather than the secondary effects to OVX induced overfeeding [33]. In another E2-deficient aromatase knockout (ArKO) mouse model, spontaneous obesity and hepatic steatosis result from impaired fatty acid *β*-oxidation and elevated fatty acid synthase (FAS) in the liver in both female and male mice [34]. These findings are further supported by previous studies demonstrating that E2 inhibits lipogenic gene expression and lipid uptake in the liver by decreasing lipoprotein lipase activity, as well as promoting lipolysis by increasing expression of hormone-sensitive lipase and adipose TG lipase in the liver [35, 36].

ER-*α* is the predominant ER subtype presented in both male and female hepatocytes [19, 20]. Estrogen signaling is important in both males and females in the regulation of lipogenesis, demonstrated by using animal models and human studies. Specifically, estrogens regulate the activity and expression of lipogenic genes to directly inhibit lipogenesis in several animal species [37, 38]. Liver enzymes may also be regulated by circulating estrogen levels. One study of genome-wide analyses demonstrated that the subtle oscillations of estrogens occurring during the estrous cycle are sufficient to influence liver gene expression, and that ERs are involved in the pulsatile synthesis of fatty acids and cholesterol in the liver [27]. Thus this study demonstrated the importance of the maintenance of estrogen oscillation to limit fat deposition in the hepatic tissues in females [27]. Additionally, treatment of the specific ER-*α* agonist PPT decreases weight, fat mass, and TG in the liver in both wild-type mice and obese* ob*/*ob* mice [39, 40]. Thus, the metabolically protective effect of estrogen may be attributed to estrogen signaling via ER-*α* [41].

This is further demonstrated by investigation of estrogen and estrogen signaling using knockout or transgenic animal models. Male and female ER-*α* knockout mice exhibit hepatic steatosis by increasing gene expression of lipogenic transcription factors such as sterol regulatory element binding protein 1c (SREBP-1c) and decreasing lipid transport genes [42, 43]. Mice with liver-specific ER-*α* knockout [44, 45] or liver-specific GPER knockout [46] show increases in fat accumulation in the liver and develop disturbed insulin signaling under high-fat diet (HFD) feeding. Thus, hepatic steatosis has been observed in both of the above genetic models, one with liver-specific ER-*α* knockout with functional GPER and the other with liver-specific GPER knockout with functional ER-*α*. Thus, although it is widely recognized that estrogens regulate liver lipid metabolism and reduce triglyceride accumulation in the liver mainly via ER-*α* [47, 48], both ER-*α* and GPER are required to be present in the liver to maintain lipid homeostasis. Estrogen is produced in males by aromatization of testosterone. Male but not female mice in which the aromatase gene has been deleted (ArKO) develop hepatic steatosis that can be normalized by estrogen treatment [49]. Thus, E2 treatment reduces fatty acid synthesis and lipid accumulation and prevented NAFLD in castrated male rats [50].

Hepatic TG and diacylglyceride increase in the livers of ER-*α* knockout male mice under HFD feeding, explained by dysregulation of insulin-stimulated ACC phosphorylation and DGAT1/2 protein levels [44]. Interestingly, a recent study using specific plasma membrane ER-*α* knockout has demonstrated that it is the membrane-localized ER-*α*, but not nuclear ER-*α*, that is responsible for protection from hyperlipidemia by decreasing expressions of many hepatic genes involved in lipid synthesis, at least in female mice with OVX [51]. Although ER-*α* is antilipogenic in the liver, the role of ER-*β* in the liver is not consistent in the literature. ER-*β* deficient mice have higher body weight but lower liver weight due to increased insulin sensitivity and decreased TG accumulation in the liver [52], indicating that ER-*β* might be lipogenic and diabetogenic in the liver. Opposite finding has been reported where, different from treatment of E2 or ER-*α* agonists that decrease hepatic PPAR*γ* expression, treatment of ER-*β* agonist 8*β*-VE2 comparably elevates PPAR*γ* expression to the same mRNA level as non-drug treated group in the liver of HFD-fed female rats with OVX [53]. Interestingly, all treatments of E2, ER-*α* agonist, or ER-*β* agonist are capable of reducing TG accumulation in the liver of HFD-fed rats with OVX [53]. Thus, the mechanism for reduced hepatic lipid accumulation in both suppressed ER-*β* signaling as seen in ER-*β* knockout mice [52] and activated ER-*β* signaling as seen in ER-*β* agonist-treated rats [53] is awaiting further elucidation. Hepatic steatosis is also found in GPER deficient female mice fed with HFD rather than male mice [46]. Although both 6-month-old female and male GPER KO mice display increased body weight, only female mice had glucose intolerance, while male mice developed glucose intolerance at the age of 18 months [54]. Furthermore, GPER agonist G-1 decreases fatty acid synthesis and TG accumulation in both human and rodent pancreatic *β* cells [55], but the effect of G-1 treatment on lipid metabolism in the liver is not clear. Both liver GPER and membrane-associated ER-*α* are critical for liver lipid metabolism. However, it is possible that GPER has greater impacts on male lipid regulation [54], whereas membrane-associated ER-*α* variant [51] may have greater impacts on female lipid regulation, as female livers have markedly higher expression of all three membrane-associated ER-*α* variants compared with male livers [56].

### 2.3. Estrogens Regulate Glucose Homeostasis in the Liver

Hepatic glucose homeostasis is determined by glucose uptake and glucose production. The major glucose transporter (GLUT) in the liver is GLUT2 that bidirectionally transports glucose across liver cell plasma membrane, efflux of glucose formed from gluconeogenesis or glycogenolysis out of liver cells, and uptake of circulating glucose into liver cells. Hepatic GLUT2 is upregulated by glucose, FAS, and insulin [57]. Since estrogen treatment has been shown to increase insulin synthesis and release [58], estrogens might indirectly increase GLUT2 expression in the liver, which has not been demonstrated yet. A recent study demonstrates that it is estriol, instead of E2, that downregulates GLUT2 in pregnant women during late stages of pregnancy whose peak postprandial glucose levels are much lower than glucose levels of healthy nonpregnant women [59]. Estrogens are also important in hepatic insulin clearance. Several lines of evidence show that intravenous conjugated estrogen treatment or low dose of oral contraceptive does not significantly alter insulin sensitivity but slightly increases hepatic insulin clearance in postmenopausal women [60, 61]. Estrogens reduce gluconeogenesis and increase glycogen synthesis and storage in the liver, lowering circulating glucose level [43, 62]. Additional observations using rodents with OVX that lacks majority of endogenous estrogens support the notion that estrogens lower glucose levels [63, 64]. A recent study reports increased glucagon signaling due to increased amount of glucagon receptor that accounts for enhanced glucose production, accompanied with increased gluconeogenic enzymes in rats with OVX [65]. Interestingly, such changes cannot be prevented by E2 replacement, which indicates that disrupted liver glucose homeostasis following OVX is not merely caused by deficiency of endogenous E2 [65] but could be caused by deficiency of other ovarian hormones such as progesterone. Although classic nuclear progesterone receptor has not been found in the liver [22, 66], progestins can either bind to membrane-bound progesterone receptors [67] or bind to ARs [22] in human liver and carry metabolic effects. On the other hand, estrogens are also found to facilitate epinephrine's action via *β*2-adrenergic receptor in regulating glycogenolysis and gluconeogenesis in the rat liver to increase circulating glucose level [68].

Estrogen signaling is important in both males and females in the regulation of glucose homeostasis, improving glucose tolerance and insulin sensitivity, demonstrated by using animal models and human studies [69–71]. Additionally, although estrogens do not affect hepatic glucose metabolism* in vivo*, estrogens increase insulin receptor to enhance glucose metabolism* in vitro* [72, 73].

ER-*α* deficient mice exhibit significantly impaired glucose tolerance and hepatic insulin resistance, while ER-*β* deficient mice exhibit normal glucose tolerance, suggesting that ER-*α* instead of ER-*β* plays an important role in the regulation of hepatic glucose homeostasis [43]. The importance of ER-*α* in the regulation of hepatic glucose tolerance is further supported by inadequate suppression of hepatic glucose production during hyperinsulinemic clamp study in ER-*α* deficient mice [74]. Although impaired glucose tolerance is seen in GPER1 knockout mice, GLUT2 and glucokinase are not affected [1], and glucose production in liver has not been measured yet. Hepatic PPAR*γ* expression rises markedly following OVX in HFD-fed rats [53]. The rats treated with E2 or ER-*α* agonist have reduced PPAR*γ* expression in the liver, whereas the rats treated with ER-*β* agonist maintain a similarly high mRNA level of PPAR*γ* as non-drug treated HFD-fed rats with OVX. The sustained hepatic PPAR*γ* gene expression correlates with increased glucose uptake into the liver of rats with OVX [53].

### 2.4. Estrogens Regulate Cholesterol Homeostasis in the Liver

Dyslipidemia is determined by decreased HDL but increased LDL and TG in the blood. The liver is the principal organ for cholesterol* de novo* biosynthesis, which is catalyzed by the rate limiting enzyme 3-hydroxy-3-methyl-glutaryl-CoA reductase (HMGR). The SREBP-1c is the master regulator of cholesterol by stimulating transcription of LDL and HMGR [75]. Postmenopausal women have elevated LDL and VLDL and lower HDL [76].

A previous* in vitro* study points out that HMGR promoter is induced by estrogen treatment in the breast cancer cell line MCF-7 but not in any hepatic cell line [77], indicating differential regulation of HMGR by estrogens among different tissues. Estrogen treatment does not increase cholesterol synthesis in liver cells* in vitro*. In an* in vivo* study using castrated male rats, DHT, but not E2, treatment increases phosphorylation of HMGR to decrease cholesterol synthesis in the liver [50]. Thus, at least in castrated male rats, androgen action is associated with downregulation of cholesterol biosynthesis in the liver.

Estrogens also decrease LDL level and increase HDL to promote cholesterol secretion into bile in postmenopausal women [78]. Total cholesterol and LDL are elevated in ArKO mice with E2 deficiency [79]. Increased hepatic HMGR activity and subsequently increased levels of cholesterol and LDL are seen in rats with OVX with reduced level of endogenous estrogens [65]. Estrogen replacement in both ArKO mice and rats with OVX normalizes the levels of LDL and cholesterol. The above mentioned cell, animal, and human studies collectively indicate important roles of estrogens in reducing LDL and increasing HDL.

ER-*α* is able to protect the liver from hypercholesterolemia [47, 48]. To support this, lack of ER-*α* (whole body) is associated with increased expression of genes involved in lipid biosynthesis and lipid metabolism [43]. A male patient without functional ER-*α* has been reported with dyslipidemia [80], supporting the importance of ER-*α* in regulating cholesterol homeostasis. Consistently, the expressions of ER-*α* (and AR) and phosphorylated HMGR are significantly reduced in the human liver samples from male severe steatotic NAFLD patients compared with the liver samples from subnormal men [50].

Aromatase deficient mice without endogenous estrogen production exhibit obesity [79] and dyslipidemia [81] and mice with liver-specific ER-*α* knockout accumulate liver triglycerides and diacylglycerides [42, 43]. In contrast, ER-*α* agonist PPT increases the expression of genes involved in lipid oxidation and metabolism [82]. Additionally, ER-*α* deficient mice and ER-*α* and ER-*β* double knockout mice display increased body fat and serum cholesterol level, but these changes are not found in ER-*β* deficient mice [83].

In GPER KO mice, LDL levels increase approximately by 200%, but HDL levels do not show any significant differences from WT, which indicates that GPER mainly regulates the LDL metabolism instead of HDL [54]. A recent study shows that human individuals with hypofunctional P16L genetic variant of GPER have increased plasma LDL [84, 85]. In contrast, GPER activation upregulates LDL receptor expression in the liver via downregulation of proprotein convertase subtilisin kexin type 9 to enhance LDL metabolism [85]. Interestingly, animals with estrogen deficiency do not increase cholesterol synthesis; instead they decrease cholesterol catabolism by reducing activity of 7*α*-hydroxylase, the enzyme that catalyzes the initial step in cholesterol catabolism and bile acid synthesis in calcium supplementation-induced hypercholesterolemia [86]. This study further demonstrates that estrogen treatment protects against increase in circulating level of cholesterol by activating of GPER [86].

## 3. The Role of Androgens in Regulating Liver Energy Homeostasis

### 3.1. Androgen Signaling

The major circulating androgens include dehydroepiandrosterone, androstenedione, testosterone, and dihydrotestosterone (DHT), in descending order of circulating concentrations. Only testosterone and DHT bind to the AR whereas the rest are considered as proandrogens. Within target cells, testosterone can be converted to active androgen DHT via 5*α*-reductase or converted to E2 by aromatase.

ARs are expressed in the liver of male and female humans and rodents, and AR expression in the liver is sex dependent. In adult rats, basal AR expression in the liver of male rats is about 20 times higher than that in the liver of female rats [87]. AR expression is also age dependent in the liver of either sex, which is very low, almost undetectable, before puberty, increases in postpubertal life, and gradually declines during aging, reaching an almost nondetectable level after about 22–24 months of age in rats [88]. The sex- and age-dependent AR expression in the liver is programed by a regulatory element in the AR gene promoter [89].

There are isoforms of ARs which are AR-A with N-terminal truncated that resulted from proteolysis and AR-B with full length [90, 91]; among these two AR isoforms, the AR-B with full length is more potent than AR-A [92]. It is not clear, however, which isoform of AR is dominant in the liver. Androgens, like estrogens, work on both nuclear and nonnuclear receptors. The genomic effect of androgens is achieved through activation of nuclear receptor, followed by binding to specific DNA known as androgen response element (ARE) motifs in its target gene [93]. AR can recruit other transcription factors such as AP-1, nuclear factor-*κ*B, sex-determining region Y, and the E26 transformation-specific family of transcription factors and bind to DNA regions other than ARE, to participate in transcription activation of many other genes [94]. The nonnuclear receptor of androgens function is independent of DNA interaction and is more rapid by interacting with cytoplasmic signal transduction pathways, including PKA and MAPK/ERK [95] (Figure 1). The AR knockout animals are well developed, but the membrane-only AR knockout animals are not established yet, and that is why the exact role of membrane AR in liver metabolism is unclear.

### 3.2. Androgens Regulate Lipid Homeostasis in the Liver

Many studies have shown that androgens and androgen signaling suppress the development of hepatic steatosis [96, 97]. One population-based cross-sectional study has reported a close association between low serum testosterone level and hepatic steatosis in men [98]. Mice with 5*α*-reductase knockout do not covert testosterone to DHT. These mice upregulate expression for the genes involved in lipid storage and downregulate genes for fatty acid oxidation and accumulate lipid in their livers when they are fed with HFD [99]. An inhibitor of 5*α*-reductase induces liver steatosis in male obese Zucker rats [100]. Therefore, normal level of active androgen is critical to prevent liver steatosis.

Besides androgen level, ARs are also critical in maintaining lipid metabolism in the liver. Testicular feminized (Tfm) mice with nonfunctional AR and very low serum testosterone levels greatly increase HFD feeding-induced hepatic lipid deposition compared with control male mice with functional AR and normal circulating levels of testosterone. Replacement of testosterone reduces lipid deposition in the liver of Tfm mice to a similar level to control males [101]. Moreover, Kelly et al. [101] found that the expressions of key regulatory enzymes for fatty acid synthesis, including acetyl-CoA carboxylase (ACC) and FAS, are elevated in placebo-treated Tfm mice comparing with placebo-treated wild-type littermates and Tfm mice receiving testosterone treatment, indicating that the action of androgens on lipid deposition is independent of AR and at least partially via affecting key regulatory lipogenic enzymes to protect against hepatic steatosis [101]. Male but not female hepatic ArKO mice fed with a normal chow diet developed liver steatosis at 10 months with reduced fatty acid oxidation and increased* de novo* fatty acid synthesis [102]. Thus, males with either functional AR or normal circulating testosterone level would maintain normal level of fatty acid synthesis and avoid increased lipid deposition in the liver.

Although many studies have shown that androgens protect against NAFLD [50, 103], other studies have reported an opposite finding that androgens promote NAFLD development and progression [104, 105]. The inconsistencies might be due to different animal models employed and different treatments utilized in various studies. The findings reported by Münzker et al. indicate that the testosterone/DHT ratio is more important for NAFLD development and progression than concentrations of testosterone and/or DHT [106]. In contrast, the role of AR in hepatic steatosis is less controversial. The total AR knockout mice develop liver steatosis and insulin resistance in both male and female mice [107]. Hepatic AR knockout mice with HFD feeding also show hepatic steatosis and insulin resistance, via upregulation of hepatic expression of SREBP-1c, ACC, and PPAR*γ* to increase lipid synthesis and downregulation of PPAR*α* to decrease fatty acid oxidation; interestingly, such effects are evident in males but absent in females [102, 108]. Thus, hepatic AR plays more critical roles in maintaining liver lipid metabolism in males than in females.

Testosterone is either converted to E2 binding to ERs or converted to DHT binding to ARs. From the above studies, ARs are vital in regulating liver lipid homeostasis in both males and females [107], although hepatic ARs have greater impact in males than in females [102, 108]. In order to test the role of androgen-AR signaling in female metabolic process, Kanaya et al. replace DHT in female mice with OVX and find that those mice accumulate greater amount of fat in the liver and develop other symptoms and signs of metabolic dysfunction when these mice are fed with either a standard chow diet or HFD [109]. Therefore, androgen action has great impact on lipid metabolism in female livers.

Women have lower basal levels of androgens compared with males, and increased androgen level can affect metabolism in women. The role of androgens in females is not well established, but many lines of evidence indicate that hyperandrogenism in women with polycystic ovary syndrome (PCOS) increases risk of developing NAFLD. NAFLD is frequently present in PCOS women with excessive production of androgens by the ovaries and thus elevated circulating level of androgens, suggesting that abnormally high level of androgens in women may contribute to increased fat storage in the liver. It is noteworthy that the risk for NAFLD in women with PCOS is independent of obesity or insulin resistance but is triggered directly by the hepatotoxic, destructive effect in the liver, indicated by elevated level of alanine aminotransferase [110]. To summarize, normal level and signaling of androgens prevent hepatic lipid accumulation in males, while androgen deficiency in males is associated with fatty liver. Abnormally high level of androgens increases lipid deposition in the liver in females. Androgens therefore have differential effects in men and women.

### 3.3. Androgens Regulate Glucose Homeostasis in the Liver

Testosterone levels are lower in diabetic men than nondiabetic men [111]. Androgen deprivation therapy for prostate cancer patients lowers their circulating testosterone level and increases their risk of diabetes [112, 113] and not only increases circulating level of glucose but also diminishes pancreatic cell function [114]. Testosterone treatment markedly reduces circulating levels of glucose and TG in men [115].

GLUT2 directionally transports glucose across liver cell plasma membrane to maintain glucose homeostasis, as mentioned above in Section 2.3. Upregulation of GLUT2 plays a more critical role in regulating glucose export out of, rather than regulating glucose import into, the liver. It has been reported that blood glucose level, along with the mRNA and protein levels of GLUT2 in the liver, significantly increases following castration in male rats with deficiency of endogenous androgens [116]. Supplementation of testosterone or a combination of testosterone with E2 normalizes GLUT2 mRNA and protein levels in the livers of castrated rats, whereas treatment of E2 alone does not have any effect [116]. These findings suggest that testosterone maintains glucose homeostasis by regulating hepatic glucose output, and testosterone deprivation due to castration increases hepatic glucose output, induces hyperglycemia, and develops symptoms seen in type 2 diabetes and metabolic syndrome. Testosterone replacement restores GLUT2 mRNA and protein levels suggesting that testosterone may have a direct effect on GLUT2 transcription and translation of mRNA. Although the presence of ARE has not been identified in the promoter region of GLUT2, AR could function as a ligand-activated transcription factor by itself [117] or bind to some other coactivators [118, 119] to increase GLUT2 expression.

In contrast, estrogens have little effect on hepatic GLUT4 and insulin receptor in male rats, but estrogens increase level of insulin receptor in HepG2, a liver cancer cell line [72]. Interestingly, insulin receptor mRNA level as well as insulin sensitivity is increased in a human liver cell line when being treated with testosterone [73]. Similarly, replacement of testosterone in castrated male mice also increases insulin receptor mRNA and protein levels in the liver and normalizes castration-induced glucose metabolic impairment [120]. Treatment of testosterone induces glycogen synthesis in both intact and castrated male rats [108, 121].

High testosterone level is associated with a low risk of diabetes in men, whereas it is associated with a high risk of diabetes in women [111, 122–124]. Excess androgen in women with PCOS impairs hepatic glucose metabolism by decreasing insulin-stimulated glucose uptake and glycogen synthesis and predisposes women with PCOS to insulin resistance [125, 126]. Metformin, the most commonly used first-line drug to treat diabetes, is found to be effective to treat NAFLD and also suppresses the serum androgen concentration in PCOS patients [127, 128]. Increased androgen activity in postmenopausal women correlates with impaired glucose tolerance [129, 130].

To summarize, testosterone in males favors hepatic glucose metabolism, whereas testosterone in females impairs it. Thus, androgens in males and females differentially regulate glucose homeostasis.

### 3.4. Androgens Regulate Cholesterol Homeostasis in the Liver

Old men have increased risks of developing dyslipidemia with increased serum cholesterol and LDL levels, and decreased HDL level, and testosterone replacement reverses such dyslipidemia [108]. Hepatic scavenger receptor class B member 1 (SR-1B) is important in regulating cholesterol uptake from circulating HDL. DHT treatment in castrated obese mice increases SR-1B compared with vehicle-treated castrated mice. At the same time, LDL secretion is decreased by DHT treatment. Cholesterol 7*α*-hydroxylase, a key enzyme in bile formation and cholesterol removal, is also decreased after DHT treatment. All these above results provide a comprehensive explanation for how chronic androgen replacement can decrease serum levels of cholesterol and LDL via enhancing liver cholesterol uptake and via suppressing cholesterol removal, which in turn increases liver cholesterol accumulation [120]. A clinical study, however, shows that a single dose of testosterone treatment increases the serum cholesterol level after two days by increasing the expression of HMGR, the rate limiting enzyme for cholesterol* de novo* biosynthesis in the liver, but 15 days after the testosterone administration the cholesterol levels in the volunteers were back to baseline levels [131]. The mechanisms for the androgen induced upregulation of HMGCR transcription as well as the physiological consequences have not been investigated and need to be further elucidated.

## 4. Summary and Future Directions

The metabolic syndrome and its related diseases, such as obesity and diabetes, increase the health problems worldwide. The liver is the largest organ in the body that regulates lipid, glucose, and cholesterol homeostasis. Hepatic steatosis is one of the major manifestations of metabolic syndrome. Several lines of epidemiological data have suggested that sex hormones are associated in fatty different types of receptors. Estrogens seem to play protective roles against hepatic fat accumulation via suppressing lipogenesis and gluconeogenesis and promoting lipolysis and glycogen storage. Interestingly, estrogens increase both cholesterol synthesis and secretion. ER-*α* and its membrane form are more important in regulating energy homeostasis than ER-*β*. GPER and its roles in energy homeostasis are currently under intensive investigation; however, there is less evidence about the role of GPER in the liver compared with classic nuclear estrogen receptors. Since the GPER specific agonist and antagonist have been developed, further studies should apply these new chemicals to examine the role of GPER in liver energy homeostasis, yet the underlying molecular mechanisms are still unclear and longing for further investigation.

**Figure 2:**
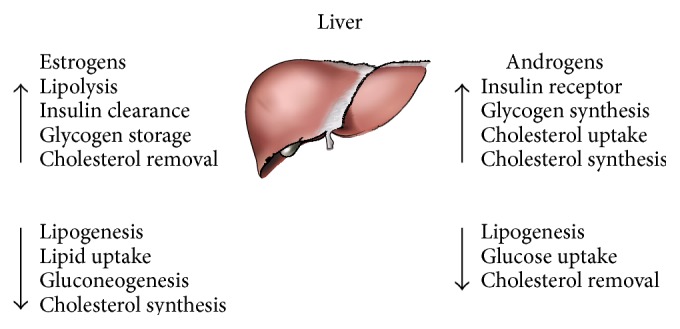
Metabolic effects of estrogens and androgens on regulation of lipid, glucose, and cholesterol in the liver.

We review and discuss the roles played by estrogens, androgens, and their receptors in regulating liver energy homeostasis (Figure 2). The action mechanisms of estrogens are complicated in the body, as they work through multiple different subtypes of estrogen receptors. Estrogens promote liver glucose storage via increasing glucose transporters and glycogen synthesis and suppress liver glucose production via decreasing gluconeogenesis. Estrogens also actively participate in maintaining lipid and cholesterol balance and play protective roles against hepatic lipid accumulation, via suppressing lipogenesis, lipid uptake, and cholesterol synthesis and promoting lipolysis and cholesterol removal. Interestingly, estrogens increase both cholesterol synthesis and secretion. Classic nuclear ER-*α* and its membrane form are more important in regulating energy homeostasis than ER-*β*. GPER and its roles in energy homeostasis are currently under intensive investigation; however, there is less evidence about the roles of GPER in the liver compared with classic nuclear ERs. Since the GPER specific agonist and antagonist have been developed, further studies should apply these new chemical compounds to examine the role of GPER in liver energy homeostasis.

Androgens and nuclear AR have been shown to increase insulin receptor, decrease lipogenesis, and promote cholesterol storage in the liver. The membrane AR, however, is not well studied, which is also a potential research area to explore. It must be emphasized that the integration of nongenomic effects via membrane receptor signaling and genomic effects via nuclear receptor signaling of sex hormones is critical to produce the final sex hormone cellular outcomes.

Further investigation about differential androgen action in males and females is needed. Androgen deficiency, or excessive androgens as seen in women with PCOS, the most common endocrine disorder and cause of infertility among women of reproductive age, is closely associated with disturbed lipid and glucose metabolism in the liver.

## Abbreviations

ARs: Androgen receptors
ARE: Androgen response element
ArKO: Aromatase knockout
DHT: Dihydrotestosterone
E2: Estradiol
ER: Estrogen receptor
ER-*α*: Estrogen receptor-*α*
ER-*β*: Estrogen receptor-*β*
ERE: Estrogen response element
FAS: Fatty acid synthase
GPER: G protein-coupled receptor
GLUT: Glucose transporter
HDL-C: High density lipoprotein cholesterol
HFD: High-fat diet
HMGR: 3-Hydroxy-3-methyl-glutaryl-CoA reductase
LDL-C: Low density lipoprotein cholesterol
NAFLD: Nonalcoholic fatty liver disease
OVX: Ovariectomy
PCOS: Polycystic ovary syndrome
PPAR*γ*: Proteasome proliferator activated receptor-*γ*
SR-1B: Scavenger receptor class B member 1
SREBP-1c: Sterol regulatory element binding protein 1c
TG: Triglyceride.
